# Impact of nutritional status in the era of FOLFOX/FIRI-based chemotherapy

**DOI:** 10.1186/s12957-017-1226-0

**Published:** 2017-08-24

**Authors:** Shunji Okada, Shintaro Yamazaki, Teruo Kaiga, Tomoya Funada, Mitsugu Kochi, Tadatoshi Takayama

**Affiliations:** Department of Digestive Surgery, Nihon University School of Medicine, 30-1 Oyaguchikami-machi, Itabashi-ku, Tokyo 173-8610 Japan

**Keywords:** Colorectal cancer, Nutrition, Chemotherapy

## Abstract

**Background:**

The nutritional status plays a pivotal role during anticancer therapy. This study analyzed whether nutritional status influences the outcomes in the era of FOLFOX/FIRI therapy.

**Methods:**

The patients were divided into two groups according to whether the nutritional status was well (serum albumin level ≥ 3.8 g/dL or a ≥ 1.0 g/dL increase as compared with the value before chemotherapy) or not before and 2 and 6 months after the start of chemotherapy. Chemotherapy-related adverse events (AE), treatment effect, and compliance were evaluated according to the nutritional status. The progression-free survival (PFS) and overall survival (OS) were assessed based on the nutritional status at 6 months.

**Results:**

Between 2010 and 2013, data on 108 consecutive patients were analyzed. At 2 months after chemotherapy, the hematotoxicic AE and the value of tumor markers did not differ significantly. The non-hematotoxic AE were less frequent in patients in the well-nourished group (grade 2, 15.9 vs. 38.5%, *p* < 0.01). Based on the nutritional status at 6 months after chemotherapy, the hematotoxicic AE (grade 3, 9 vs. 19.5%, *p* = 0.03) and non-hematotoxic AE (grade 2, 31.3 vs. 51.2%, *p* = 0.04; grade 3, 6.0 vs. 24.4%, *p* < 0.01) were less frequent, and the median CEA value (5.3 vs. 27.75 mg/L, *p* < 0.01) was significantly lower in the well-nourished group. The median PFS (364 vs. 233 days, *p* < 0.01) and 5-year OS (26.5 vs. 11.1%, *p* = 0.01) are significantly better in the well-nourished group.

**Conclusions:**

The well-nourished at initial 6 months may predict a better treatment response and fewer adverse events in FOLFOX/FIRI chemotherapy.

## Background

Innovations in combination chemotherapy regimens (FOLFOX/FORFIRI plus targeted agents) for colorectal metastasis have facilitated the control of tumor progression in patients with unresectable disease [1–3]. Consequently, the survival of patients with unresectable colorectal cancer has been extended [4, 5].

Tumor progression due to a poor response to chemotherapy or worsening of general condition caused by treatment-related toxicity is the main reasons for discontinuing chemotherapy [6]. Although most clinical studies have focused on the effects of chemotherapy, the nutritional status of patients during chemotherapy is also an important factor [1–3]. Cancer-related malnutrition is multifactorial and depends primarily on disease course and general condition of the patient.

Recently, nutritional support has been shown to enhance the response to anticancer treatment, and early nutritional support contributes to patients’ survival [7–9]. However, little information is available regarding the relation between the nutritional status and the outcomes of chemotherapy. The aim of this study was to analyze whether nutritional status influences the occurrence of adverse events, continuance of chemotherapy, and survival in the era of FOLFOX/FORFIRI therapy.

## Methods

### Participants

The study group comprised patients who received chemotherapy (FOLFOX/FOLFIRI with or without an anti-vascular endothelial growth factor antibody or an anti-epidermal growth factor receptor agent) for initially unresectable or recurrent colorectal cancer. All patients were able to have perioral food intake. When ileus was observed, transient colostomy was made to have perioral food intake as much as possible. Patients were excluded if they had received adjuvant chemotherapy, double primary cancers, and severe comorbidity requiring a reduction in the dose of chemotherapy. The data was retrospectively collected after the chemotherapy.

### Surgical indication for colorectal metastasis

The indication for liver resection was the presence of entire tumors of colorectal origin, which if removed would be curative in the absence of any medical contraindications. Neither the number of tumors nor the tumor diameter limited the indication for liver resection [10]. The upper limit of the liver volume to be resected was assessed by the indocyanine green retention rate at 15 min. If the estimated remnant liver volume was considered insufficient, portal vein embolization was performed before the liver resection [11]. The presence of extrahepatic metastases did not contraindicate liver resection if the lesion was estimated to be curable by metachronous resection.

### Assessments

The patients were divided into two groups according to whether the nutritional status was well or bad-nourished, assessed before chemotherapy and 2 months (after initial 4 cycles) and 6 months after the chemotherapy. Well-nourished was defined as a serum albumin level of ≥ 3.8 g/dL or an increase in the serum albumin level of ≥ 1.0 g/dL as compared with the baseline value before chemotherapy (3.8 g/dL: lower limit of normal value at our institution). The control group was defined as a serum albumin level of < 3.8 g/dL or a decrease in the serum albumin level of < 1.0 g/dL when the baseline serum albumin level was ≥ 3.8 g/dL.

Chemotherapy-related adverse events (hematotoxic AE, non-hematotoxic AE, discontinue or dose reduction of scheduled chemotherapy) were evaluated 2- and 6 months after the chemotherapy. Based on the nutritional status at 6 months after the start of chemotherapy, the response of chemotherapy, progression-free survival (PFS), and overall survival (OS) were evaluated.

Hematotoxicity was assessed according to the Common Terminology Criteria for Adverse Events (CTC-AE), version 5.0. [12] Complications were assessed and graded by a single observer (S.O.) who was not involved in the administration of chemotherapy. This study was confirmed by the institutional review board of our hospital.

### Statistical analysis

Data are expressed as median values and ranges or as absolute values and percentages. *p* values of < 0.05 were considered to indicate statistical significance. Student’s *t* test, the *χ*2 test, the Mann-Whitney *U* test, and the Fisher’s exact test were used for univariate analysis as required. Survival rates were calculated using a Cox proportional-hazards model, survival curves were obtained using the Kaplan-Meier method, and comparisons were made using the log-rank test. All statistical analyses were performed with the use of a statistical software package (JMP version 9.0, SAS Institute Inc., Cary, NC, USA).

## Results

### Participants

Between May 2010 and January 2013, FOLFOX/FOLFIRI-based chemotherapy was given to 157 consecutive patients with colorectal cancer. A total of 49 patients were excluded: 31 patients had no target lesions (adjuvant chemotherapy), 9 had double primary cancers, 9 had serious concurrent illnesses requiring reduced doses of anticancer agents, and 1 received cetuximab. Data on the remaining 108 consecutive patients were analyzed.

### Patient characteristics before chemotherapy

There was a total of 108 patients and the median age was 65 years (range 34–83) (Table 1).

**Table 1:** Patient characteristics

			*n* = 108
Age	Median (range)		65 (34–83)
Gender (male/female)			58/50
Performance status	(0, 1, 2, 3)		(91, 16, 1, 0)
Body mass index^a^			23.3 (13.7–36.9)
Primary tumor site			
	Colon/rectum		75/33
	Right-sided/left-sided		51/57
Residual of primary tumor			17 (15.7%)
History of liver resection for metastasis			27 (25.0%)
Number of liver metastasis^a^			5 (0–28)
Target lesion of chemotherapy			
	Primary tumor		19 (17.6%)
	Lymph node		28 (25.9%)
	Peritoneum		18 (16.7%)
	Lung		29 (26.9%)
	Liver		43 (39.8%)
KRAS status	Wild		67 (62.0%)
First line chemotherapy	FOLFOX/FOLFIRI		30/78
Add-on targeting agent	VEGFR		41 (38.0%)
	EGFR		38 (35.1%)
	None		29 (26.9%)
Preoperative status^a^			
	White blood cell	(mm^3^)(mm^3^)	6000 (3400–9950)
	Neutrophil	(mm^3^)(mm^3^)	3887 (1659–3750)
	Lymphocyte	(mm^3^)(mm^3^)	1352 (512–2195)
	Albumin	(g/dl)	3.8 (2.4–4.7)
	Total bilirubin	(mg/dl)	0.46 (0.19–3.82)
	Aspartate aminotransferase	(IU/L)	23 (12–203)
	Lactate dehydrogenase	(IU/L)	196 (21–1949)
	Blood urea nitrogen	(mg/dL)	13.7 (4.7–27.7)
	Creatinine		0.68 (0.42–1.59)
	C-reactive protein	(mg/dL)	0.45 (0.1–8.94)
	Carcinoembryonic antigen	(mg/L)	18.0 (4.7–65.3)
	CA19-9	(mg/L)	35.7 (6.5–129.4)

Data express median with internal quadorant range

^a^Median with range

There were 75 patients with colon cancer and 33 patients with rectal cancer. The 17 patients had a primary unresectable cancer with metastasis. The rate of KRAS gene mutation is 38.0% (41/108 patients). There were 30 patients (27.8%) of FOLFOX and 78 patients (32.2%) of FOLFIRI at first line chemotherapy. The 41 patients (38.0%) received anti-vascular endothelial growth factor (VEGFR) antibody, and the 38 patients (35.1%) received an anti-epidermal growth factor receptor antibody. The serum carcinoembryonic antigen (CEA) value (median 18.0 ng/mL [interquartile range (IQR): 4.7–65.3]) and the carbohydrate antigen (CA) 19-9 level (35.7 U/mL [6.-329.4]).

### Hematotoxicity and nutritional status at 2 months

The nutritional status was estimated and divided into the two groups according to the nutritional status after the 4 cycles (2 month) of FOLFOX/FIRI therapy. There were 69 patients in the well-nourished group and 39 patients in the control group (Table 2). The proportion of age, gender, WHO performance status, BMI, site of primary tumor, and residual of the tumor did not differ significantly between the well-nourished and control. There was no significant difference of the first line chemotherapy regimen and additional targeting agent between the two groups.

**Table 2:** Serum albumin trends at 2 months after chemotherapy

	Well-nourished (*n* = 69)	Bad-nourished (*n* = 39)	*p* value
Age (median, range)	63 (34–83)	66 (36–80)	0.67
Gender (male)	38	20	0.7
WHO performance status (> 2)	6 (8.7%)	11 (28.2)	< 0.01
Body mass index (median, range)	24.1 (18.6–33.8)	23.1 (13.3–28.8)	0.47
Primary site			
Rectum	23	10	0.4
Right-sided (*n* = 51)	27	24	0.03
Left-sided (*n* = 57)	40	17	0.15
Residual of primary tumor	12	5	0.53
First line regimen			
FOLFOX/FOLFIRI	19/56	11/22	0.94
Add-on targeting agent			
VEGFR (*n* = 41)	30 (43.5%)	11 (28.2%)	0.11
EGFR (*n* = 38)	26 (37.7%)	12 (30.8%)	0.47
None (*n* = 29)	13 (18.8%)	16 (41.0%)	0.01
Adverse event^a^			
Hematotoxicity			
Grade 2	13 (18.8%)	12 (30.8%)	0.11
Grade 3	4 (5.8%)	4 (10.3%)	0.39
Grade 4	0 (0%)	1 (2.6%)	0.18
Non-hematotoxicity			
Grade 2	11 (15.9%)	15 (38.5%)	< 0.01
Grade 3	7 (10.1%)	6 (15.4%)	0.42
Grade 4	1 (1.4%)	0 (0%)	0.68
Tumor marker^b^			
CEA	16.8 (3.7–45.1)	6.9 (2.9–14.4)	0.39
CA19-9	19.6 (2.3–46.9)	15.6 (6.9–81.2)	0.19

^a^According to the CTC-AE Ver5.0

^b^Median with internal quadorant range

As for the chemotherapy-induced AE, the hematotoxic AE tended to less frequent in well-nourished (grade 2 18.8 vs. 30.8%, *p* = 0.11). The non-hematotoxic AE was significantly less frequent in well-nourished (grade 2 15.9 vs. 38.5%, < 0.01). There was no significant difference in the median tumor marker value in 2 months after initial chemotherapy (CEA 16.8 vs. 6.9 mg/L, *p* = 0.39; CA19-9 19.6 vs. 15.6, *p* = 0.19).

### Patients’ outcomes and nutritional status at 6 months

Six months after the start of chemotherapy, there were 67 patients in the well-nourished and 41 in the control group (Table 3). There were significant difference in the proportion of bad WHO performance status (> 2) [6 (9.0%) patients vs. 16 (39.0%) patients, *p* < 0.01] and who were able to have perioral intake [67 (100%) patients vs. 36 (87.9%) patients, *p* = 0.02]. The median number of cycles of performing FOLFOX/FIRI therapy during 6 months was significantly longer in the well-nourished group than that in the bad-nourished group [21 cycles, (6–24) vs. 11 (4–24), *p* < 0.01]. As for the AE of the chemotherapy, the hematotoxic AE was significantly less frequent in well-nourished (grade 3, 9.0 vs. 19.5%, *p* = 0.03). The non-hematotoxic AE was also significantly less frequent in patients with well-nourished (grade 2, 31.3 vs. 51.2%, *p* = 0.04, grade 3, 6.0 vs. 24.4%, *p* < 0.01). The rate of discontinue of chemotherapy (6.0 vs. 34.1%, *p* < 0.01) and dose reduction (6.0 vs. 24.4%, *p* < 0.01) was significantly smaller in patients in the well-nourished group. The rate of conversion surgery is significantly frequent in the well-nourished (9.0 vs. 2.4%, *p* = 0.18). The median serum CEA level was significantly lower in the well-nourished than in the control group (5.3 U/mL [2.85–15.05] vs. 27.75 U/mL [7.98–78.9], *p* < 0.01), while the CA19-9 value did not differ significantly (*p* = 0.25).

**Table 3:** Serum albumin value at 6 months after chemotherapy

		Well-nourished (*n* = 67)	Bad-nourished (*n* = 41)	*p* value
Age	(Median, range)	64 (35–80)	68 (34–83)	0.23
Gender		37	21	0.68
WHO performance status	(> 2)	6 (9.0%)	16 (39.0)	< 0.01
Body mass index^a^	(Median, range)	24.1 (17.9–35.0)	23.1 (20.1–28.8)	0.67
Perioral food intake		67 (100%)	36 (87.9%)	0.02
Primary site				
Rectum		22	18	0.25
Right-sided	(*n* = 51)	26	24	0.04
Left-sided	(*n* = 57)	44	13	< 0.01
Residual of primary tumor		13	4	0.18
Continuing regimen				
FOLFOX/FOLFIRI		16/51	13/29	0.38
Performing chemotherapy cycles during 6 months		21 (6–24)	11 (4–24)	< 0.01
Add-on targeting agent				
VEGFR	(*n* = 48)	34 (50.7%)	14 (34.1%)	0.09
EGFR	(*n* = 26)	11 (16.4%)	15 (36.6%)	0.02
None	(*n* = 34)	22 (32.8%)	12 (29.3%)	0.7
Adverse event				
Hematotoxicity				
	Grade 2	21 (31.3%)	20 (48.8%)	0.18
	Grade 3	6 (9.0%)	8 (19.5%)	0.03
	Grade 4	0 (0%)	1 (2.4%)	0.72
Non-hematotoxicity				
	Grade 2	21 (31.3%)	21 (51.2%)	0.04
	Grade 3	4 (6.0%)	10 (24.4%)	< 0.01
	Grade 4	1 (1.5%)	1 (2.4%)	0.73
Discontinue^b^		4	14	< 0.01
Dose reduction		4	10	< 0.01
Conversion to surgical treatment		6	1	< 0.18
Tumor marker^c^				
CEA (mg/L)		5.3 (2.85–15.05)	27.75 (7.98–78.9)	< 0.01
CA19-9 (mg/L)		15 (7.05–71.25)	33.3 (6.15–1040.13)	0.25

^a^Available 89 patients’ data

^b^Except for conversion surgical treatment patients

^c^Median with internal quadorant ratio

### Progression-free survival and overall survival based on the nutritional status

**Fig. 1:**
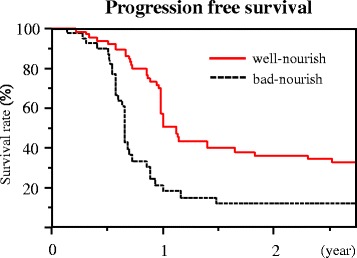
Progression-free survival according to the nutritional status 6 months after chemotherapy. The median progression-free survival was significantly longer in the well-nourished group than in the bad-nourished group (median, 333 vs. 242 days, *p* = 0.03)

**Fig. 2:**
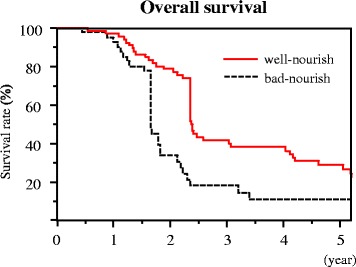
Overall survival according to the nutritional status 6 months after chemotherapy. The overall survival rate was significantly higher in the well-nourished group than in the bad-nourished group (1 year, 95.7 vs. 80.0%; 2 years, 80.2 vs. 47.1%; and 3 years, 60.8 vs. 37.7%; *p* = 0.01, respectively)

The median follow-up time in this study is 3 years. The median PFS is significantly better in the well-nourished than that in the control (364 vs. 233 days, *p* < 0.01) (Fig. 1). Also, the OS is significantly better in the well-nourished (1 year, 96.9 vs. 92.7%; 3 years, 38.2 vs. 18.4%; 5 years, 26.5 vs. 11.1%, *p* = 0.01) (Fig. 2).

## Discussion

Our study showed that patient’s nutritional status during chemotherapy is closely related to the occurrence of AE and response of chemotherapy. The well-nourished at 6 months after chemotherapy is associated with a better response to anticancer therapy, and it contributes to prolong PFS and OS in FOLFOX/FIRI therapy.

Malnutrition is a serious problem in patients who receive anticancer therapy. Cancer-related malnutrition is multifactorial and reflects the balance between disease course and its treatment [4–6, 13]. Nutritional imbalance is caused by tumor progression and cancer-related hyper metabolism [8, 9, 12]. Chemotherapy-induced adverse events also negatively affect nutritional status [14]. Our study showed that the nutritional status at initial 2 months might be very important to continue chemotherapy without severe AE. Therefore, nutritional support may indispensable for the patients in the bad-nourished group.

The hematotoxic AE more objectively reflects chemotherapy-induced damage, and it often predicts the occurrence of severe patients’ condition such as sepsis. In contrast, the non-hematotoxic AE reflects an early sign of bad-nourished during chemotherapy. Our study showed that the non-hematotoxic AE occurred prior to the hematotoxic AE. Therefore, maintaining patients well-nourished during chemotherapy might have a key role in the outcomes of treatment. Therefore, the measurement of serum albumin trend may be important to predict patient’s outcomes.

A low serum albumin level before surgery is known to be associated with poor outcomes in patients with colorectal cancer. The Glasgow prognostic score, a combined score based on hypoalbuminemia and the C-reactive protein level, is an established predictor of survival after surgery [8, 9]. Information on the nutritional status of patients who receive chemotherapy remains limited. Our results were consistent with the Glasgow prognostic score and suggested that nutritional status during chemotherapy might play a vital role in outcomes.

The trend in serum CEA levels, PFS, and OS was better in the well-nourished. The patients in the well-nourished group completed chemotherapy as scheduled without serious adverse events. Therefore, there was no need for dose reduction and pending of chemotherapy in patients in the well-nourished group. Consequently, a sufficient response to treatment was obtained, and general condition will improve as the results of anticancer therapy.

## Conclusion

In conclusion, maintaining a well-nourished during FOLFOX/FIRI therapy might contribute to higher response to cancer and fewer adverse events for patients. A nutritional support should be one of the options for the patients in bad-nourished such as highly advanced cancer and older patients.
